# Associations of the Seed Fatty Acid Composition of Sesame (*Sesamum indicum* L.) Germplasm with Agronomic Traits and *FAD2* Variations

**DOI:** 10.3390/plants13121590

**Published:** 2024-06-07

**Authors:** Eun-Gyeong Kim, Sookyeong Lee, Tae-Jin Yang, Jae-Eun Lee, Eunae Yoo, Gi-An Lee, Jungsook Sung

**Affiliations:** 1 National Agrobiodiversity Center, National Institute of Agricultural Sciences, Rural Development Administration, Jeonju 54874, Republic of Korea; keg950@korea.kr (E.-G.K.); xsanta7@korea.kr (S.L.); jnlee88@korea.kr (J.-E.L.); eung77@korea.kr (E.Y.); 2 Department of Agriculture, Forestry and Bioresources, Plant Genomics and Breeding Institute, Research Institute of Agriculture and Life Science, College of Agriculture and Life Sciences, Seoul National University, Seoul 08826, Republic of Korea; tjyang@snu.ac.kr

**Keywords:** sesame, oleic/linoleic acid ratio, agronomic traits, genotype, *FAD2*

## Abstract

Sesame is an important oilseed crop grown for human consumption in many countries, with a high commercial value due to its high oleic/linoleic acid ratio (O/L ratio). However, its properties may vary among different accessions. In the current study, 282 sesame accessions were evaluated to determine the effects of agronomic traits and genotypes on the O/L ratio. The O/L ratio was positively correlated with the oleic acid (C18:1), stearic acid (C18:0), and myristic acid (C14:0) concentrations, as well as the capsule zone length (CZL), capsule width (CW), and capsule length (CL), and negatively correlated with the linoleic acid (C18:2) and linolenic acid (C18:3) concentrations, the days to maturity (DTM), days to flowering (DTF), and the height of the first capsule-bearing node (HFC) (*p* < 0.05). In addition, the O/L ratio was affected by the *FAD2* haplotype, as the Hap2 and Hap3 sesame accessions had lower O/L ratios. Therefore, we suggest that the increase and decrease in the contents of C18:1 and C18:2 are associated with the *FAD2* haplotype. A total of 25 agronomic traits and fatty acid compositions were compared via statistical analysis, and accessions with a high O/L ratio were selected. The results of this study can be used as a basis for further research on the development of new sesame varieties through enhancing nutritional functionality.

## 1. Introduction

Sesame (*Sesamum indicum* L.), one of the oldest crops cultivated by humans [1], was first cultivated in Babylon and Assyria and continues to be used today for a variety of purposes, including as a seasoning and food [2]. Sesame seeds are rich in nutritional benefits. Notably, they are abundant in vitamin E, an essential antioxidant that helps protect cells from damage [3]. Additionally, sesame seeds contain linoleic acid, which helps reduce cholesterol production and supports heart health. Beyond these, sesame seeds serve as a valuable source of fiber, calcium, and plant-based protein, making them a nutritious addition to any diet [4]. Sesame is also one of the world’s most important oilseed crops due to its relatively high oil content [5], which typically exceeds 50% of the seed weight [6]. Sesame seed oil is highly stable due to the presence of chemicals with high natural antioxidant activity, such as sesamolin, sesamin, and sesamol [7]. The consumption of sesame products can lower the serum lipid concentration and improve the antioxidant status in people with hyperlipidemia [8]. Additionally, sesamol may be used as a potential cancer preventive agent [9]. More than 80% of the fatty acids in sesame oil are oleic and linoleic acids [6], and the high content of these monounsaturated and polyunsaturated fatty acids improves the oil quality, which is beneficial for human consumption [10].

Fatty acid composition is influenced by both genetic and environmental factors, making it an important variable that determines seed quality and oil properties [11]. The genotype–environment interaction (GEI) plays a significant role in this, whereby a genotype may be well adapted to one environment but not to another. Previous research indicates that, under drought-stress conditions, sesame varieties with a high oleic acid content have a decreased oleic acid content and an increased linoleic acid content [12,13]. This local adaptability leads to a variation among genotypes, resulting in different GEIs for various accessions [14]. For instance, genetic studies of sesame have shown strong genetic control over the oil content and fatty acid composition [15]. Therefore, securing accessions with diverse genotypes is a priority if we are to reveal associated factors. Accordingly, in this study, we used 282 accessions, including cultivars, breeding lines, and landraces originating from 31 countries, as experimental materials.

Genes that affect the fatty acid composition of sesame have been identified. *FAD2*, a fatty acid desaturase (FAD), plays a crucial role in this process by converting oleic acid (C18:1) to linoleic acid (C18:2) through the addition of a double bond at the 12-carbon position of the hydrocarbon chain [16]. Functional mutations in the *FAD*2 enzyme can either decrease or increase *FAD* activity, leading to corresponding changes in the levels of C18:1 and C18:2. Consequently, different *FAD2* genotypes can significantly influence the level of C18:1 in sesame seeds [17]. C18:1 is a monounsaturated acid and constitutes the major fatty acid in sesame. The consumption of crop oil with a high C18:1 content, typically around 70–80%, may offer health benefits by reducing the risk of cardiovascular disease by approximately 15% [18]. Particularly, a high ratio of oleic to linoleic acid can enhance the marketability of sesame by rendering it more resistant to rancidity and extending the shelf life of high-quality seeds and related products [19]. Therefore, selecting accessions with a high O/L ratio remains one of the primary objectives in sesame breeding.

The objectives of this study were to analyze the effects of agronomic traits and genotype on the O/L ratio of sesame seeds, to determine the nucleotide diversity in the coding region of *FAD2*, and to identify sesame accessions with a high O/L ratio that may offer health benefits. The findings of this study will provide valuable insights for future molecular breeding programs and studies focusing on the utilization of functional genes in sesame.

## 2. Results

### 2.1. Variation in Traits among 282 Sesame Accessions

**Figure 1:**
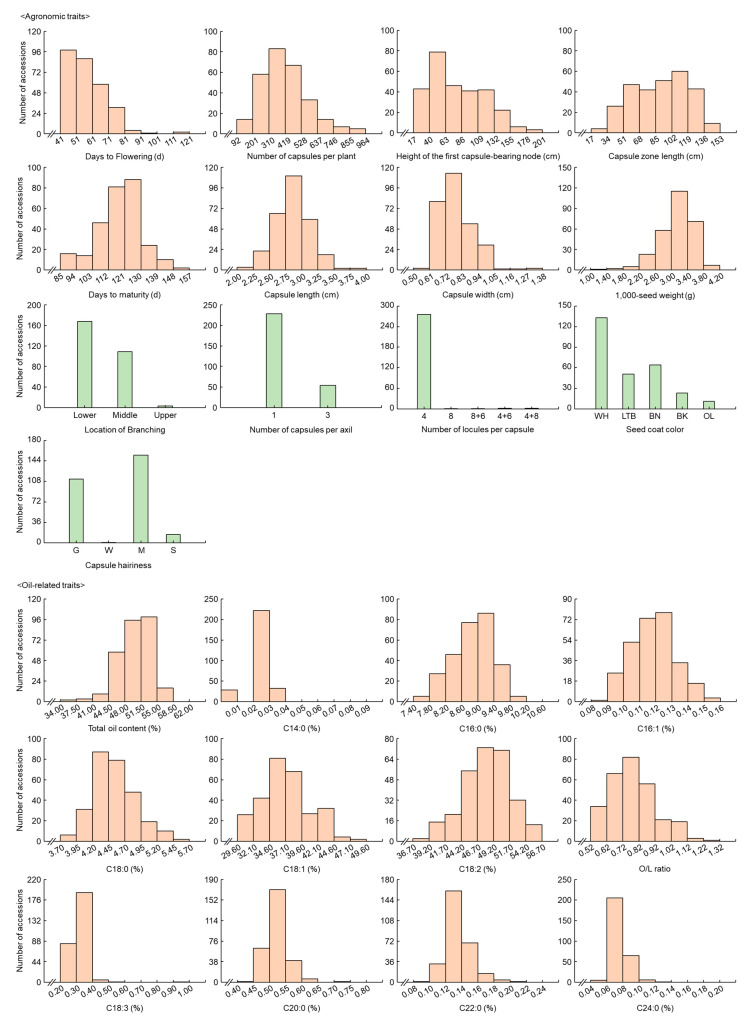
Variation in agronomic and oil-related traits among the 282 sesame accessions. This figure displays histograms representing the distribution of data values for each trait. Quantitative traits include agronomic traits such Days to flowering (DTF), number of capsules per plant (NCP), height of the first capsule-bearing node (HFC), capsule zone length (CZL), days to maturity (DTM), capsule length (CL), capsule width (CW), and 1000-seed weight (1000-SW), as well as oil-related traits including total oil content (TOC), and fatty acid composition (myristic [C14:0], palmitic [C16:0], palmitoleic [C16:1], stearic [C18:0], oleic [C18:1], linoleic [C18:2], oleic/linoleic ratio [O/L ratio], linolenic [C18:3], arachidic [C20:0], behenic [C22:0], lignoceric [C24:0]). Qualitative traits include location of branching (LB), number of capsules per axil (NCPA), number of locules per capsule (NLPC), seed coat color (ScC), and capsule hairiness (CH), with specific categories such as NCPA (1: single capsule, 3: three capsules), NLPC (4: four locules, 8: eight locules, 8 + 6: a mixture of 8 and 6 locules, 4 + 6: a mixture of 4 and 6 locules, and 4 + 8: a mixture 4 and 8 locules.), ScC (WH: white, LTB: light brown, BN: brown, BK: black, OL: olive), and CH (G: glabrous, W: weak, M: medium, S: strong). Orange bars indicate quantitative traits, while green bars represent qualitative traits.

The traits of the investigated sesame accessions are depicted in Figure 1. The frequency distribution of these traits is shown in Table S2.

Agronomic traits such as DTF (days to flowering), NCP (number of capsules per plant), HFC (height of the first capsule-bearing node), CZL (capsule zone length), DTM (days to maturity), CL (capsule length), CW (capsule width), and 1000-SW (1000-seed weight), along with oil-related traits including TOC (total oil content), C14:0 (myristic acid), C16:0 (palmitic acid), C16:1 (palmitoleic acid), C18:0 (stearic acid), C18:1 (oleic acid), C18:2 (linoleic acid), O/L ratio (oleic/linoleic ratio), C18:3 (linolenic acid), C20:0 (arachidic acid), C22:0 (behenic acid), and C24:0 (lignoceric acid), exhibited wide variation and normal distributions. Additionally, qualitative agronomic traits such as LB (location of branching), NCPA (number of capsules per axil), NLPC (number of locules per capsule), ScC (seed coat color), and CH (capsule hairiness) showed wide variation.

### 2.2. Correlation and Principal Component Analysis (PCA) of Agronomic Traits and Oleic/Linoleic Ratio in Sesame Accessions

**Figure 2:**
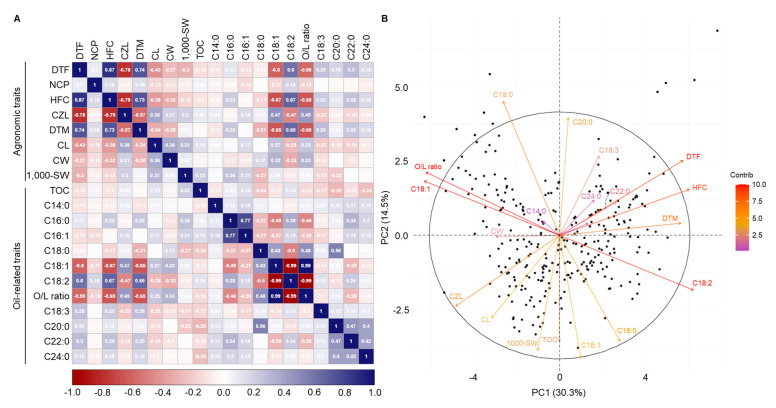
Correlation and PCA analysis of agronomic traits and oil-related traits. (**A**) Correlation of agronomic traits and oil-related traits. Positive and negative correlations between traits are indicated by purple and red backgrounds, respectively. The correlation is stronger when the hue is darker. Correlation analysis was performed using the corrplot function from the ggcorrplot package of the R program. (**B**) PCA analysis of agronomic traits and oil-related traits. The redder the color of the directional lines, the greater their contribution to the factor map projected onto PC1 and PC2. Directional lines that are more vertical indicate a lesser correlation between the principal components and each trait. PCA was analyzed using the fviz_screeplot from the FactoMineR package of the R program.

A Pearson correlation analysis was conducted on quantitative agronomic traits, including DTF, NCP, HFC, CZL, DTM, CL, CW, and 1000-SW, as well as oil-related traits such as TOC, C14:0, C16:0, C16:1, C18:0, C18:1, C18:2, O/L ratio, C18:3, C20:0, C22:0, and C24:0 (Figure 2A). All data were analyzed at the 5% significance level, and correlation coefficients (r) are presented accordingly. The O/L ratio was positively correlated with the CZL (r = 0.45), C18:1 (r = 0.99), and C18:0 (r = 0.48). The O/L ratio was negatively correlated with C16:0 (r = −0.46), C18:2 (r = −0.99), DTM (r = −0.59), and HFC (r = −0.65).

To evaluate the relationship between agronomic traits and the O/L ratio, we performed a PCA on the entire dataset (Figure 2B and Table S3). Twenty principal components (PCs) were obtained. PC1 (30.3%), PC2 (14.5%), PC3 (11.3%), PC4 (5.6%), PC5 (5.1%), PC6 (4.6%), and PC7 (4.5%) collectively explained 75.8% of the total trait variation (Table S3). Based on PC1, the O/L ratio, C18:1, C18:0, C14:0, and CW exhibited consistent directions, while the DTF, HFC, DTM, C22:0, C24:0, C18:3, and C20:0 showed different directions (Figure 2B). TOC and C20:0 demonstrated low correlations, suggesting their limited representation in the PC1 factor map. On the other hand, based on PC2, the O/L ratio, C18:1, and C18:0, showed consistent directions, while C18:2, C16:0, and C16:1 exhibited opposite directions. CW, C14:0, C22:0, and C24:0 showed weaker correlations based on the PC2 criterion, indicating their lesser representation in the PC2 factor map. The projection of variables onto the two-component factor map highlighted the significant contributions of the O/L ratio, C18:1, DTF, HFC, DTM, CZL, and C18:2. These seven factors were categorized into four groups: (i) the O/L ratio and C18:1; (ii) DTF, HFC and DTM; (iii) C18:2, and (iv) CZL. Consequently, the O/L ratio exhibited positive correlations with C18:1, C18:0, CZL, and CW while showing negative correlations with C18:2, DTM, HFC, DTF, and LB.

**Figure 3:**
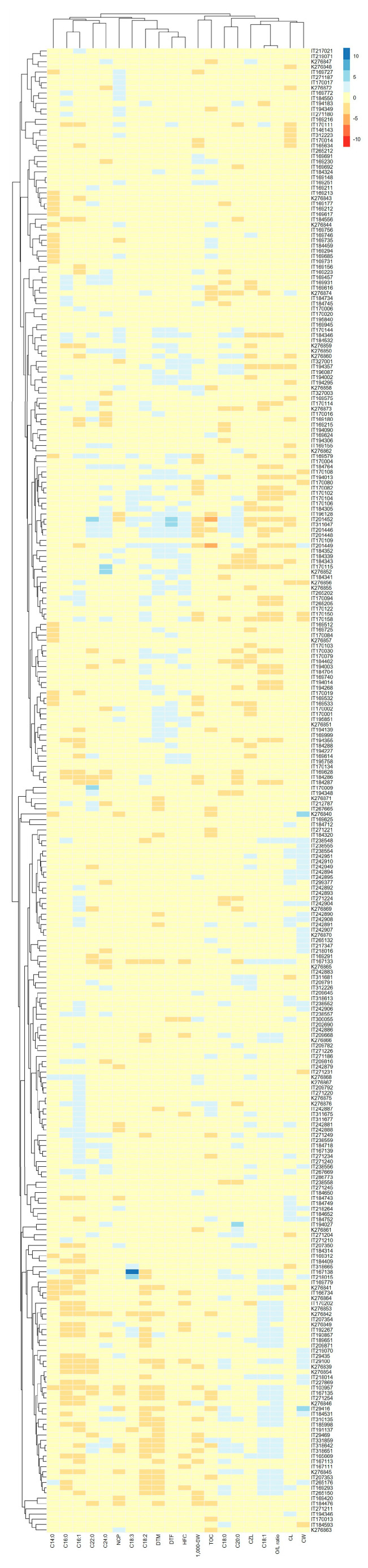
Heatmap for screening based on the correlation between O/L ratio and agronomic traits in 282 sesame accessions. This heatmap displays screening of accessions with high or low O/L ratios, consistent with the results from the correlation analysis between agronomic traits and the O/L ratio. Correlation analysis among sesame accessions was performed using the pheatmap package of the R program (Version 4.3.2).

The correlation analysis examined the relationships between various traits and the O/L ratio (Figure 3). Based on their correlations with the O/L ratio and their contributions, 23 sesame accessions (IT029100, IT029416, IT103957, IT167133, IT169293, IT207353, IT209668, IT218015, IT265176, IT271254, K276845, IT310135, IT318642, IT318651, IT331859, IT201446, IT201448, IT201449, IT201452, K276873, K276874, IT184346, and IT194357) were selected for further study (Table S4). The fifteen accessions with high O/L ratios were IT029100, IT029416, IT103957, IT167133, IT169293, IT207353, IT209668, IT218015, IT265176, IT271254, K276845, IT310135, IT318642, IT318651, and IT331859. In these accessions, the O/L ratio was positively correlated with C18:0 and C18:1 and negatively correlated with C18:2 and DTM. The eight accessions with low O/L ratios were IT184346, IT194357, IT201446, IT201448, IT201449, IT201452, K276873, and K276874. In these accessions, the O/L ratio was positively correlated with C18:1 and negatively correlated with C18:2, C20:0, DTF, DTM, and HFC.

### 2.3. Genetic Architecture Variation of the O/L Ratio in Sesame Accessions

**Figure 4:**
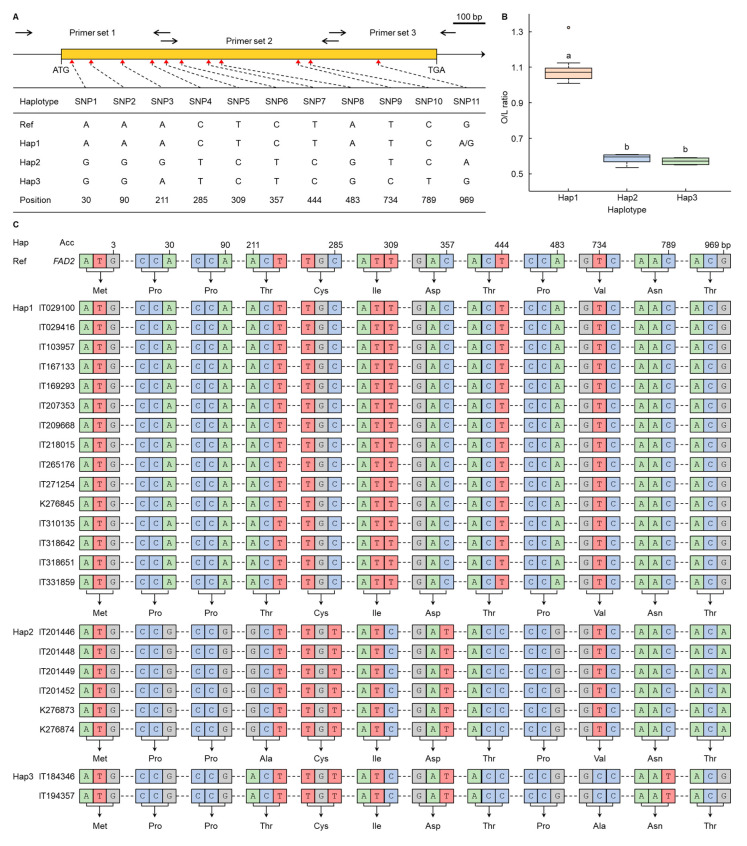
Haplotype analysis of *FAD2* in sesame accessions. (**A**) A total of 11 SNPs were identified in the *FAD2* sequence of sesame accessions. (**B**) The O/L ratio of the haplotypes present in sesame accessions is presented as a boxplot. The same letter in a column is not significantly different at the 5% level by the Duncan Multiple Range Test (DMRT). (**C**) The DNA sequence and corresponding synthesized amino acids for each haplotype of the screened accessions are shown.

In this study, the O/L ratios of a total of 282 sesame accessions were screened. From these, 60 accessions representing the top 10% and bottom 10% of the O/L ratio range were initially selected. These accessions were then further analyzed using correlation analysis, principal component analysis (PCA) for each trait, and Pearson correlation among the accessions. Based on their correlations with the O/L ratio and their contributions, 23 sesame accessions were finally selected for haplotype analysis of the *FAD*2 region (Figure 4). The haplotype analysis identified three haplotypes, with a total of 11 single nucleotide polymorphisms (SNPs) observed (Figure 4A). Compared to the reference sequence (NCBI No. AY770501), Hap1 exhibited a substitution from G to A/G at position 969 bp in the coding sequence of *FAD2*. Hap2 displayed substitutions from A to G at positions 30, 90, and 211 bp, followed by a C to T substitution at 285 and 357 bp and a T to C substitution at 309 and 444 bp. Additionally, Hap2 showed substitutions at 483 bp from A to G and at 969 bp from G to A. Hap3 had substitutions from A to G at positions 30 and 90 bp, from C to T at positions 285, 357, and 789 bp, and from T to C at positions 309, 444, and 734 bp. Furthermore, a substitution at 483 bp from A to G was observed in Hap3. The O/L ratio of sesame accessions with Hap1 in the *FAD2* sequence ranged from 1.01 to 1.32 (Figure 4B), while those with Hap2 displayed ratios ranging from 0.53 to 0.60. Similarly, sesame accessions with Hap3 demonstrated ratios ranging from 0.55 to 0.59. ANOVA and post-hoc (DMRT) analyses showed that the O/L ratio of Hap1 was significantly higher than that of Hap2 and Hap3 (Table S5). Notably, the substitution of A to G at 211 bp in Hap2 resulted in the synthesis of alanine (Ala) instead of threonine (Thr) in the protein (Figure 4C). Similarly, a C to T substitution at 734 bp in Hap3 led to the synthesis of Ala instead of valine (Val). SNP3 and SNP9 were responsible for changes in protein synthesis, while the remaining SNPs resulted in the synthesis of the same protein as the reference sequence.

## 3. Discussion

Many factors affect the fatty acid content of oil, such as the growing conditions, maturity, and sesame genotype. In the current study, we investigated the properties of 282 sesame accessions to analyze the correlation between the O/L ratio and various agronomic traits, including DTF, NCP, HFC, CZL, DTM, CL, CW, 1000-SW, TOC, C14:0, C16:0, C16:1, C18:0, C18:1, C18:2, O/L ratio, C18:3, C20:0, C22:0, and C24:0. The O/L ratio was positively correlated with C18:1, CZL, CW, CL, C18:0, C14:0, C20:0, and 1000-SW, and negatively correlated with C18:3, C18:2, DTM, DTF, HFC, C16:0, C16:1, C22:0, C24:0, NCP, and TOC. Similarly, in Slover and Lanza [20], the O/L ratio of sesame was positively correlated with C18:1 and CZL and negatively correlated with C18:2, DTM, DTF, and HFC. This suggests that the O/L ratio of sesame is associated with C18:1, C18:2, DTF, DTM, HFC, and CZL.

In the principal component analysis (PCA), PC1 (30.3%) and PC2 (14.5%) collectively explained 44.8% of the total trait variation. At the PC1 level, the O/L ratio exhibited a positive correlation with C18:1, indicating they are oriented in the same direction. At the same time, C18:2 showed a negative correlation with the O/L ratio, implying they are oriented in opposite directions. Conversely, at the PC2 level, the O/L ratio aligned with C18:1, C18:0, etc., while DTF and HFC aligned in different directions. Moreover, C18:2 exhibited an opposite direction to the O/L ratio. Additionally, significant contributions were observed from the O/L ratio, C18:1, DTF, DTM, HFC, and C18:2 at both the PC1 and the PC2 levels. Previous research reported a negative correlation between the O/L ratio, C18:2, and DTF [21], while in [22], a negative correlation between the O/L ratio and HFC was noted. Consequently, it is anticipated that early-maturing cultivars with earlier DTF and DTM values, along with a shorter HFC, will have an enhanced O/L ratio. The use of sesame accessions with high O/L ratios and early maturing cultivars could serve as a valuable strategy to mitigate extreme drought and high-temperature stress during the growing season.

The correlation and PCA analysis identified 282 sesame accessions whose O/L ratios were positively correlated with C18:1, C18:0, CW, CH, and CL and negatively correlated with C18:2, DTM, DTF, and HFC. Among these, accessions with both high and low O/L ratios were selected. The accessions with high O/L ratios included IT029100, IT029416, IT103957, IT167133, IT169293, IT207353, IT209668, IT218015, IT265176, IT271254, K276845, IT310135, IT318642, IT318651, and IT331859, whereas those with low O/L ratios comprised IT184346, IT194357, IT201446, IT201448, IT201449, IT201452, K276873, and K276874. The countries of origin for each selected sesame accession were as follows: IT029100, IT029416, IT109357, IT265176, IT271254, IT310135, IT318642, IT318651, and IT331859 were from Korea (KOR). IT167133 and IT218015 were from Bulgaria (BGR). IT169293, IT207353, K276845, and IT276845 were from the Greece (GRC). IT209668 was from Russia (RUS). On the other hand, IT184346 and IT194357 originated from Ethiopia (ETH), and IT201446, IT201448, IT201449, and IT201452 were from the Philippines (PHL). K276873 and K276874 were from the Kenya (KEN). It is noteworthy that accessions with low O/L ratios predominantly originated from countries with higher average temperatures, mostly located near the equator. This observation aligns with [23], which suggests that O/L ratios decrease under high-temperature stress.

The *FAD2* sequences of 23 selected sesame accessions were compared. For the 15 sesame accessions with high O/L ratios (IT029100, IT029416, IT103957, IT167133, IT169293, IT207353, IT209668, IT218015, IT265176, IT271254, K276845, IT310135, IT318642, IT318651, and IT331859), the *FAD2* sequences were identical to the reference sequence, classified as Hap1. Conversely, the accessions with low O/L ratios, namely, IT201446, IT201448, IT201449, IT201452, K276873, and K276874 were classified as Hap2. Hap2 exhibited nine single nucleotide polymorphisms (SNPs), with SNP9 being a T to C substitution at position 734 of the *FAD2* sequence, resulting in Val to Ala protein synthesis. Similarly, among the accessions with low O/L ratios, IT184346 and IT194357 were categorized as Hap3. Hap3 also displayed nine SNPs, with SNP3 being substituted from A to G at position 211 of the *FAD2* sequence, leading to protein synthesis from Thr to Ala. While Hap1 showed a wide variation in O/L ratio values, with some genotypes exhibiting high values and others showing low values, suggesting that the O/L ratio is influenced by both genotype and various environmental conditions, Hap2 and Hap3 consistently exhibited relatively low O/L ratios compared to the reference gene (accession number, AY770501). Given the alteration in protein synthesis, it was assumed that the function of *FAD2* was also modified. Specifically, in Hap2, the 211–213 bp codon was translated to Ala instead of Thr, while in Hap3, the 733–735 bp codon was translated to Ala instead of Val. In [24], it was reported that the overexpression of *FAD* in soybean increased the Ala content as well as the C18:2 content in sesame under the control of the seed-specific 2S albumin promoter. Consequently, it was determined that the protein synthesis modification accelerated *FAD2* activation. We also compared the hydrophobicity index of each amino acid, as reported by Monera et al. [25], which is a measure of how soluble an amino acid is in water. Using values normalized to assign a value of 100 to the most hydrophobic residue relative to glycine, which is considered neutral among amino acids, Val is classified as very hydrophobic (hydrophobicity index 79), Ala is hydrophobic (hydrophobicity index 47), and Thr (hydrophobicity index 13) is neutral. Hap2 was assumed to have reduced hydrophobicity due to the change in amino acid synthesis from Val to Ala, and Hap3 was assumed to have increased hydrophobicity due to the change in synthesis from Thr to Ala. It was assumed that the difference in the number of amino acids synthesized would change the polarity of the protein, which in turn would change the fatty acid content synthesized. Chen et al. [26] discovered variations in the *FAD2* gene sequence across seven accessions within the Sesamum genus, including five *Sesamum indicum* and two *Sesamum radiatum*. They found that most of the variations were between *S. indicum* and *S. radiatum*. However, our study identified variations in the *FAD2* gene within *S. indicum* alone and also observed changes in the oleic/linoleic (O/L) ratio attributable to these variations. This finding highlights the novelty of our study, providing new insights into the genetic diversity within a single species of the Sesamum genus.

The accessions with a high O/L ratio identified in this study can serve as valuable materials for producing high-quality oil products with excellent functional and storage properties. Additionally, the statistical data used to examine the relationship between the O/L ratio and each agronomic trait can be utilized as a checklist for selecting superior sesame accessions in future breeding programs.

## 4. Materials and Methods

### 4.1. Sesame Accession Cultivation and Material Preparation

Seeds from 282 sesame accessions (cultivars, breeding lines, and landraces) were sown and cultivated on 13 May 2021 in the experimental field (35°49′53.76″ N, 127°3′50.4″ E) of the National Agrobiodiversity Center (NAC), Rural Development Administration (RDA), in Jeonju, South Korea. The seeds were harvested at full maturity and dried in a drying oven (VS-1202D) at 50 °C for 3 days. For each sesame accession, 1000 harvested seeds were weighed and stored in seed envelopes at −20 °C.

### 4.2. Characterization of Agronomic Traits

The sesame accessions were characterized in terms of their agronomic traits following the methods described in [27]. Thirteen agronomic traits were investigated (Table S1), including the days to flowering (DTF), number of capsules per plant (NCP), height of the first capsule-bearing node (HFC), capsule zone length (CZL), days to maturity (DTM), location of branching (LB), number of capsules per axil (NCPA), number of locules per capsule (NLPC), seed coat color (ScC), capsule hairiness (CH), capsule length (CL), capsule width (CW), and 1000-seed weight (1000-SW). For the DTF, we examined the number of days from the sowing date until flowing was initiated in 50% of plants. The DTM measured the number of days from the sowing date until 75% of plants reached physiological maturity. The NCPA measured the number of capsules per axil extending from the stem. This involved categorizing the number of capsules into three groups: those with a single capsule, those with three capsules, and those with a combination of one and three capsules. The NLPC investigated the number of locules within each capsule. This process involved categorizing capsules based on the number of locules, including four, six, and eight locules, as well as mixtures of 4 and 6, 4 and 8, 6 and 8, and all three: 4, 6, and 8 locules. The NCPA and NLPC randomly selected ten plants and screened a representative score of them. The HFC indicated the average length from the base to the first capsule-bearing node in five plants. The CZL showed the average length from the first capsule-bearing node to the last capsule-bearing node in five plants. The NCP indicated the total number of capsules borne on a plant. The CL indicated the average capsule length of five randomly selected capsules from the middle of the main stem. The CW was the average width of five randomly selected capsules from the middle of the main stem. The 1000-SW was the weight in grams of 1000 random seeds taken from the bulk harvest. The values for NCP, CL, CW, and 1000-SW were represented as the averages obtained from ten random repetitions of measurements. The color variation of ScC was assessed based on white, gray, light brown, brown, brick red, bright black, black, and olive. The CH was assessed based on glabrous (hair absent) plants, weak or sparse, medium, and strong or profuse. The LB was accessed based on the starting point of branching from the bottom, which was the lower, middle, and upper part of the plant. The ScC, CH, and LB were each investigated from ten randomly selected plants per accession, and representative values were screened from them. All data for each trait are presented as the mean ± standard deviation.

### 4.3. Reagent Chemicals

The reagent chemicals used in this study, including sodium hydroxide, anhydrous sodium sulfate, n-hexane, 14% boron trifluoride–methanol (BF3-methanol), chloroform, methanol, and fatty acid standards (myristic acid, palmitic acid, palmitoleic acid, stearic acid, oleic acid, linoleic acid, α-linoleic acid, arachidic acid, behenic acid, and lignoceric acid), as well as HPLC analytical-grade water, were purchased directly from Sigma Aldrich (St. Louis, MO, USA). All reagent chemicals were of analytical grade and were used without further purification.

### 4.4. Determination of Total Oil Contents from Sesame Seeds

The total oil content was determined using an adapted version of a previously reported method [28]. For the extraction of total oil from sesame seeds, the Soxhlet apparatus (Soxtec^TM^ 8000 system; FOSS Tecator AB, Hillerod, Denmark) was utilized. Sesame seeds were ground, and 1 g of seed powder was mixed with 50 mL of n-hexane and placed in an extraction system set at 130 °C. The boiling, rinsing, and recovery times for extraction were 30, 60, and 20 min, respectively. The extracted oil was cooled to room temperature and then weighed. The total oil content was calculated as a percentage of the weight of the oil obtained over the weight of the extracted seed sample. Three replicates were performed for each accession to obtain the total oil content.

### 4.5. Determination of Fatty Acid Composition from Sesame Seeds

The method of fatty acid extraction from sesame accession seeds was adapted from [29]. Samples and standards were initially derivatized. Each sample tube containing 50 μL of crude fat was treated with 2 mL of NaOH to transmethylate the fatty acids and vortexed for 5 s. This was followed by heating in an 80 °C water bath for 10 min and cooling to room temperature for 5 min. To each sample tube, 2 mL of 14% cold boron trifluoride–methanol was added, and the sample was vortexed for 5 s. It was then heated in an 80 °C water bath for 10 min and cooled at room temperature for 5 min. Then, 7 mL of n-hexane and 2 mL of H_2_O were added, and the sample was vortexed for 10 s. Then, the mixture was centrifuged at 3600 rpm at 4 °C for 10 min. The n-hexane (supernatant) was collected and poured onto filter paper lined with anhydrous sodium sulfate powder and filtered. The filtrate was transferred to sample vials for gas chromatography and stored at −20 °C.

The fatty acid methyl esters (FAMEs) were analyzed using the GCMS-QP2010 Ultra Gas Chromatography instrument (Shimadzu Co., Kyoto, Japan) equipped with an HP-INNOWAX column (0.25 mm × 30 m, 0.25 μm; Agilent Technologies Inc., Santa Clara, CA, USA). During the analysis, the column was initially set at 100 °C, and the temperature was increased at a rate of 60% of the total reaction time from 100 to 170 °C and held at 170 °C for 1 min. The temperature was increased from 170 to 240 °C at a rate of 6.5% of the total reaction time and held at 240 °C for 1 min. The temperatures of the injector and detector were both set to 250 °C. The injection mode was set to split with a volume of 10 µL, and helium (He) was used as the carrier gas. The flow pressure of the carrier gas was 117 kPa, the purge flow rate was 1.5 mL/min, and the split ratio was 50. The fatty acids were identified using related standards, and the peak areas were used to quantify the percentage of total fatty acids.

### 4.6. Screening Sesame Accessions

Traits positively correlated with the O/L ratio in accessions with high contributions were screened. These included C18:1, C18:0, CZL, CW, CL, C14:0, C20:0, and 1000-SW. Conversely, traits negatively correlated with the O/L ratio were also considered, and accessions with high contributions in this regard were selected. These traits included C18:2, DTM, DTF, HFC, C16:0, C16:1, C22:0, C18:3, C24:0, NCP, and TOC. Additionally, accessions with low O/L ratios and positive correlations with traits making high contributions were screened. Further screenings focused on accessions with negative correlations, namely, C18:1, C18:0, CZL, CW, CL, C14:0, C20:0, and 1000-SW, and those with high contributions were finally selected.

### 4.7. Haplotype Classification of FAD2 from Sesame Accessions

All samples were obtained from 282 sesame accessions conserved in the National Genebank of the Accessions Center. To analyze the sequence divergence of *FAD2* in sesame accessions, genomic DNA was extracted from 14-day-old sesame leaves using the Gentra Puregene Tissue Kit (QIAGEN, Inc., Valencia, CA, USA). *FAD2* was amplified by PCR using genomic DNA as a template. Three primer sets were utilized to synthesize the FAD2 coding sequence (CDS) region into three fragments. Each primer set is represented by the following sequences:

Primer set 1: Forward (F): 5′-TTTTTCGGGTGTTCGTTACC-3′, Reverse (R): 5′-GCCACTGGTAATCGCTGAAT-3′.

Primer set 2: F:5′-GCTACCTAGCTTGGCCCATT-3′, R: 5′-AGAGCTCCCCTTAGCCAGTC-3′.

Primer set 3: F: 5′-TGGGGTACCGTTACTCATTG-3′, R: 5′-AGAATCCGACCAGCACAGAC-3′.

Primer set The PCR composition and conditions were as follows: PCR mix solution containing 10 ng of template DNA (10 ng/µL), 10 µL of 2X Direct buffer (inclone), 1 µL of forward and reverse primer mix, with ddH_2_O, added to make the total volume up to 20 µL. The PCR conditions were divided into three stages: stage 1—95 °C for 5 min; stage 2—95 °C for 10 s, 58 °C for 30 s, and 72 °C for 1 min, repeated 32 times; stage 3—72 °C for 5 min. We commissioned a sequencing company to analyze the *FAD2* sequences of the amplified PCR products. The three amplified sequences were assembled using Seqman software (SeqMan NGen, DNASTAR version 11; Madison, WI, USA). The sequencing data from the sesame accessions were aligned with the reference *FAD2* sequence (NCBI No. AY770501, 3762 bp, CDS: 1152 bp) using Bioedit software (version 7.2.3, Ibis Biosciences, Carlsbad, CA, USA). The sequences were analyzed to determine the haplotypes present among the sesame accessions.

### 4.8. Statistical Analysis

In this experiment, all data obtained from repeated investigations were represented as the mean ± standard deviation (SD). An analysis of variance (ANOVA), Pearson correlation, and Principal Component Analysis (PCA) were conducted using R software (Version 4.3.2, The R Foundation for Statistical Computing Platform, RStudio, Boston, MA, USA). A correlation analysis between traits was performed using the corrplot package, while PCA was analyzed using the factoextra, fviz_screeplot, and FactoMineR packages.

## 5. Conclusions

In this study, the agronomic traits and O/L ratios of 282 sesame accessions were evaluated, and a significant variation was observed in various traits. The O/L ratio was higher under conditions of higher C18:1 and C18:0 contents, longer CZL, the presence of CH, longer CL, wider CW, lower C18:2 and C18:3 content, lower LB levels, shorter DTM and DTF, and shorter HFC. In addition, three haplotypes were present, among which accessions with lower O/L ratios showed different haplotypes from the reference *FAD2*. An increase in the C18:1 content and a decrease in the C18:2 content were also associated with the *FAD2* haplotype. Based on the statistical analysis of the relationship between each agronomic trait and the O/L ratio, we provided evidence that could be important for future research and the production of seeds with high commercialization value. The results of this study can be used as an important guide for selecting sesame seeds with health benefits and can contribute to research on the development of new cultivars with a high O/L ratio.

## Data Availability

Data are contained within the article and Supplementary Materials. The authors responsible for the distribution of data and materials integral to the findings presented in this article in accordance with the policy described in the Instructions for Authors are: Gi-An Lee (gkntl1@korea.kr), Jungsook Sung (sjs31@korea.kr).

## Supplementary Materials

The following supporting information can be downloaded at https://www.mdpi.com/article/10.3390/plants13121590/s1: Table S1: List of the 282 sesame accessions used in this study; Table S2: Distribution of agronomic trait and fatty acid content data from 282 sesame accessions; Table S3: Eigenvalues and proportion of seven principal components to 20 traits of 282 sesame accessions; Table S4: Correlation between the O/L ratio and agronomic traits in 23 screened sesame accessions; and Table S5: Analysis of Variance (ANOVA) of the O/L ratio across *FAD2* haplotypes in sesame accessions.
