# Supplementary material for: Associations of the Seed Fatty Acid Composition of Sesame (Sesamum indicum L.) Germplasm with Agronomic Traits and FAD2 Variations
Source: Plants (Basel). 2024 Jun 7;13(12):1590. doi: 10.3390/plants13121590 (PMC11207932; doi:10.3390/plants13121590)
Supplement: Supplementary file 1 [file plants-13-01590-s001.zip › plants-2928865-supplementary.pdf]

Supplementary Materials

Table S1. List of the 282 sesame accessions used in this study.

Num.	Accession No.	Accession name	Origin of Country
1	IT29100	Gyeonggi Yangpyeong-1984-29100	KOREA
2	IT29416	Jeonbuk Namwon-1984-29416	KOREA
3	IT29435	Jeonbuk Muju-1984-29435	KOREA
4	K276839	IT29452-1	KOREA
5	K276840	IT29452-2	KOREA
6	IT29469	Jeonbuk Wanju-1984-29469	KOREA
7	IT103957	Jeonnam Jangseong-1985-3957	KOREA
8	IT146143	Tainan White Sesame	TAIWAN
9	K276841	IT156371-1	KOREA
10	K276842	IT156371-2	KOREA
11	IT165634	Local 41-2	EGYPT
12	IT165669	Oro Short	ITALY
13	IT166734	WHITE MUKSEOGA 2-A	TAIWAN
14	IT167111	1966 6	BULGARIA
15	IT167113	DELCO A	UNITED STATES
16	IT167133	S.M.N 21	BULGARIA
17	IT167135	VITRAGON LIGHT BROWN	BULGARIA
18	IT167138	KUBANEC 55	RUSSIAN FEDERATION
19	IT167139	TASKENYATSKIJ	RUSSIAN FEDERATION
20	K276843	IT167147-1	KENYA
21	K276844	IT167147-2	KENYA
22	IT169148	GIZA 25	EGYPT
23	IT169155	H68-21	EGYPT
24	IT169156	H71-9	EGYPT
25	IT169177	B50	EGYPT
26	IT169180	LOCAL 139	EGYPT
27	IT169211	Local 140	EGYPT
28	IT169212	Local 145	EGYPT
29	IT169213	Local 162	EGYPT

30	IT169215	Local 168	EGYPT
31	IT169216	Local 202	EGYPT
32	IT169223	B1	EGYPT
33	IT169230	B35	EGYPT
34	IT169251	Ilocos Norte Black	PHILIPPINES
35	K276845	IT169289-1	GREECE
36	K276846	IT169289-2	GREECE
37	IT169291	Rodos 37	GREECE
38	IT169293	White SINDIAos 381(e)	GREECE
39	IT169294	Kostantza 449(med.)	GREECE
40	IT169312	PI154301	MEXICO
41	IT169420	PI170755	TURKEY
42	IT169457	PI175309	INDIA
43	IT169512	PI182991	INDIA
44	IT169532	PI186509	NIGERIA
45	IT169533	PI186510	NIGERIA
46	IT169575	PI207665	JAPAN
47	IT169579	PI209965	ETHIOPIA
48	IT169614	PI226567	ETHIOPIA
49	IT169616	PI227253	IRAN
50	IT169617	PI229667	ARGENTINA
51	IT169624	PI231036	MOZAMBIQUEAMBIQUE
52	IT169625	PI231038	MOZAMBIQUEAMBIQUE
53	IT169628	PI234427	TAIWAN
54	IT169685	PI238991	GREECE
55	IT169691	PI238997	GREECE
56	IT169692	PI238998	GREECE
57	IT169725	PI249677	MOZAMBIQUE
58	IT169727	PI249679	MOZAMBIQUE
59	IT169731	PI250099	EGYPT
60	IT169735	PI250103	EGYPT
61	IT169740	PI250577	EGYPT
62	IT169746	PI250749	IRAN

63	IT169756	PI250944	IRAN
64	IT169772	PI254699	NICARAGUA
65	IT169779	PI254710	ARGENTINA
66	IT169931	PI319380	MEXICO
67	IT169945	PI343816	IRAN
68	IT169999	PI433873	NIGERIA
69	IT170001	PI433875	NIGERIA
70	IT170002	PI433876	NIGERIA
71	IT170004	PI433878	NIGERIA
72	IT170006	PI433880	NIGERIA
73	IT170009	PI433884	NIGERIA
74	IT170013	PI433890	NIGERIA
75	IT170014	PI433891	NIGERIA
76	K276847	IT170015-1	NIGERIA
77	K276848	IT170015-2	NIGERIA
78	IT170016	PI433893	NIGERIA
79	IT170017	PI433894	NIGERIA
80	IT170019	PI433896	NIGERIA
81	IT170020	PI436594	CHINA
82	IT170030	PI481879	SUDAN
83	IT170079	PI490077	SUDAN
84	IT170080	PI490078	SUDAN
85	IT170082	PI490080	SUDAN
86	IT170084	PI490082	SUDAN
87	K276849	IT170088-1	SUDAN
88	K276850	IT170088-2	SUDAN
89	IT170094	PI490094	SUDAN
90	IT170102	PI490104	SUDAN
91	IT170103	PI490105	SUDAN
92	IT170104	PI490106	SUDAN
93	IT170106	PI490109	SUDAN
94	IT170108	PI490111	SUDAN
95	IT170109	PI490112	SUDAN

96	IT170111	PI490114	SUDAN
97	IT170114	PI490118	SUDAN
98	IT170115	PI490119	SUDAN
99	IT170122	PI490127	SUDAN
100	K276851	IT170125-1	SUDAN
101	K276852	IT170125-2	SUDAN
102	IT170134	PI490140	SUDAN
103	IT170144	PI490155	SUDAN
104	IT170150	PI490161	SUDAN
105	IT170158	PI490171	SUDAN
106	IT170202	Kobayashi No.76 BON	JAPAN
107	K276853	IT170256-1	VENEZUELA
108	K276854	IT170256-2	VENEZUELA
109	IT184286	Inamar	NICARAGUA
110	IT184287	Ometepe	NICARAGUA
111	IT184288	Sabaco	NICARAGUA
112	IT184305	HU No.1991-2048	PHILIPPINES
113	IT184314	HU No.1991-2059	PHILIPPINES
114	IT184320	Local-180	EGYPT
115	IT184324	H-60-3-1	EGYPT
116	IT184339	Local 78	ETHIOPIA
117	IT184341	HU No.1991-2087	ETHIOPIA
118	IT184343	HU No.1991-2089	ETHIOPIA
119	IT184346	HU No.1991-2093	ETHIOPIA
120	IT184352	Criollo	MEXICO
121	IT184409	VENEZUELAezuela/J	VENEZUELA
122	K276855	IT184437-1	SOMALIA
123	K276856	IT184437-2	SOMALIA
124	IT184459	Greece/C	GREECE
125	IT184462	Greece/J	GREECE
126	IT184476	INDIAia/S	INDIA
127	IT184531	QAN-43	JAPAN
128	IT184532	QAN-45	JAPAN

129	IT184550	MEXICOico/E	MEXICO
130	IT184556	VENEZUELAezuela 51/D	MOZAMBIQUE
131	IT184593	No.3286	TURKEY
132	IT184650	DSS23	RUSSIAN FEDERATION
133	IT184652	TashKENYAtski No.22	RUSSIAN FEDERATION
134	IT184704	HU No.1991-2478	SUDAN
135	IT184712	PERUu	PERU
136	IT184718	HU No.1992-3023	INDIA
137	IT184734	HU No.1992-3235	VENEZUELA
138	IT184743	HU No.1992-3401	GREECE
139	IT184745	HU No.1992-3443	EGYPT
140	IT184749	HU No.1992-3489	UNITED STATES
141	IT184752	HU No.1992-3552	RUSSIAN FEDERATION
142	IT184764	HU No.1992-3805	SUDAN
143	IT185998	Jeonnam Damyang-1994-3659	KOREA
144	IT189651	Gangwon Pyeongchang-1995-1419	KOREA
145	IT191137	Chungbuk Boeun-1995-2905	KOREA
146	IT192267	Gyeongbuk Yeongil-1995-4035	KOREA
147	IT193857	Hybrid of black	KOREA
148	IT194002	VENEZUELASI00078	SENEGAL
149	IT194003	VENEZUELASI00079	SENEGAL
150	IT194013	LOCAL SESAME M-84	SUDAN
151	IT194014	LOCAL DE TAIWAN	TAIWAN
152	K276857	IT194017-1	SUDAN
153	K276858	IT194017-2	SUDAN
154	IT194027	CIANO 16	UNITED STATES
155	K276859	IT194077-1	UNITED STATES
156	K276860	IT194077-2	UNITED STATES
157	IT194090	NEBRASKA 68 S.1A	UNITED STATES
158	IT194139	DESCONOCIDA 1	UNITED STATES
159	IT194183	VENEZUELASI00284	UNITED STATES
160	IT194227	VENEZUELASI00330	UNITED STATES
161	IT194268	VENEZUELASI00373	UNITED STATES

162	IT194295	VENEZUELASI00400	UNITED STATES
163	IT194306	CA35-15	ECUADOR
164	IT194346	LATURKEY-1(TURKEY 88-A)	COLOMBIA
165	IT194348	LATURKEY-3	COLOMBIA
166	IT194349	LATURKEY-6	COLOMBIA
167	IT194355	ICA AMBALA	COLOMBIA
168	IT194357	VENEZUELASI01295	ETHIOPIA
169	IT195758	VENEZUELASI00416	UNITED STATES
170	IT195840	VENEZUELASI00502	UNITED STATES
171	IT195851	REED LIOP	JAPAN
172	IT196087	DESCONOCIDA 9	MEXICO
173	K276861	IT196113-1	INDIA
174	K276862	IT196113-2	INDIA
175	IT196128	TIPO IP 29	INDIA
176	IT201446	ACC24	PHILIPPINES
177	IT201448	ACC26	PHILIPPINES
178	IT201449	ACC28	PHILIPPINES
179	IT201452	GUIMARAS STRAIN	PHILIPPINES
180	IT202690	Local-NamaNIGERIAn	UZBEKISTAN
181	K276863	IT207348-1	GREECE
182	K276864	IT207348-2	GREECE
183	IT207350	Aspa-1452	GREECE
184	IT207353	WIR1619	GREECE
185	IT207354	Zolt Sadousei	BULGARIA
186	IT209645	UZBEKISTAN-1998-806990	RUSSIAN FEDERATION
187	IT209668	UZBEKISTAN-1998-807013	RUSSIAN FEDERATION
188	IT209782	UZBEKISTAN-1998-807127	BULGARIA
189	IT209791	UZBEKISTAN-1998-807136	BULGARIA
190	IT209792	UZBEKISTAN-1998-807137	BULGARIA
191	IT209816	UZBEKISTAN-1998-807161	BULGARIA
192	K276865	IT209824-1	RUSSIAN FEDERATION
193	K276866	IT209824-2	RUSSIAN FEDERATION
194	IT209871	UZBEKISTAN-1998-807216	RUSSIAN FEDERATION

195	IT212787	Jeju-KHC-1999-1	KOREA
196	IT217021	SALIT	ETHIOPIA
197	IT217347	Hangtian 1 hao zhima	CHINA
198	IT218014	Do-khan-tsi-ma HINDIAi	SUDAN
199	IT218015	SUNITED STATESm byal N 857	BULGARIA
200	IT218016	Jun-66	TURKEY
201	IT218264	587240W/n	KENYA
202	IT219070	587246 W/n	KENYA
203	IT219071	587303 W/n	KENYA
204	IT227869	UZBEKISTAN-UzRIPI-2001-119	COLOMBIA
205	IT238548	UzRIPI 1402	KENYA
206	IT238552	UzRIPI 1465 (brown)	CHINA
207	IT238554	UzRIPI 1470	UZBEKISTAN
208	IT238555	UzRIPI 1471	CHINA
209	IT238556	UzRIPI 1472	INDIA
210	IT238557	UzRIPI 1473	SUDAN
211	IT238558	UzRIPI 1474	SUDAN
212	IT238559	UzRIPI 1477	JAPAN
213	IT242879	UzRIPI 2	UZBEKISTAN
214	IT242881	UzRIPI 7	UZBEKISTAN
215	K276867	IT242882-1	UZBEKISTAN
216	K276868	IT242882-2	UZBEKISTAN
217	IT242883	UzRIPI 84	UZBEKISTAN
218	IT242886	UzRIPI 123	UZBEKISTAN
219	IT242887	UzRIPI 138	TURKEY
220	IT242888	UzRIPI 147	UZBEKISTAN
221	IT242890	UzRIPI 158	TURKEY
222	IT242891	UzRIPI 162	TURKEY
223	IT242892	UzRIPI 163	TURKEY
224	IT242893	UzRIPI 187	TURKEY
225	IT242894	UzRIPI 191	TURKEY
226	IT242895	UzRIPI 192	GREECE
227	IT242904	UzRIPI 267	CHINA

228	IT242906	UzRIPI 280	TURKEY
229	IT242907	UzRIPI 283	TURKEY
230	IT242908	UzRIPI 322	INDIA
231	IT242910	UzRIPI 384	UZBEKISTAN
232	IT242949	UzRIPI 1382	KENYA
233	K276869	IT242950-1	KENYA
234	K276870	IT242950-2	KENYA
235	IT242951	UzRIPI 1396 (Dark brown)	KENYA
236	IT265132	K-433	ITALY
237	IT265150	JP 213374	KOREA
238	IT265176	Kang San	KOREA
239	IT265202	Escoba blanca	PARAGUAY
240	IT265205	Kemagro 1	PARAGUAY
241	IT265212	Shirosawa	PARAGUAY
242	IT267665	THAILANDILAND-LSY-2000-29	THAILAND
243	IT267669	UzRIPI 155	TURKEY
244	K276871	IT271179-1	KENYA
245	K276872	IT271179-2	KENYA
246	IT271180	587405W/n	KENYA
247	K276873	IT271181-1	KENYA
248	K276874	IT271181-2	KENYA
249	IT271186	C.V. 74-198	VENEZUELA
250	IT271187	C.V. 74-42	VENEZUELA
251	IT271204	THAILANDILAND-JSH-2008-81024	THAILAND
252	IT271210	UzRIPI 1399	KENYA
253	IT271211	UzRIPI 1400	KENYA
254	IT271220	UzRIPI 127	TURKEY
255	IT271221	UzRIPI 141	TURKEY
256	IT271224	UzRIPI 181	TURKEY
257	IT271226	UzRIPI 188	TURKEY
258	IT271231	UzRIPI 204	GREECE
259	IT271234	UzRIPI 289	IRAN
260	IT271240	UzRIPI 546	UZBEKISTAN

261	IT271245	VENEZUELAezuela	PERU
262	IT271249	YSS-04001	INDIA
263	IT271254	Gyeonggi Yeoju-2013-32	KOREA
264	IT286773	UzRIPI 469	UZBEKISTAN
265	IT299377	UzRIPI 324	ITALY
266	IT300055	Chorbazisky	BULGARIA
267	IT310135	SD 2776	KOREA
268	IT311647	CHINA-YAAS-2010-59	CHINA
269	IT311675	UzRIPI 139	TURKEY
270	IT311677	UzRIPI 159	TURKEY
271	IT311681	UzRIPI 290	IRAN
272	IT312223	Oro	MEXICO
273	IT312226	CV 74-60	VENEZUELA
274	K276875	IT312227-1	VENEZUELA
275	K276876	IT312227-2	VENEZUELA
276	IT318613	C.V. 74-133	VENEZUELA
277	IT318642	SD 4142	KOREA
278	IT318651	SD 4466	KOREA
279	IT318665	SD 5167	KOREA
280	IT327001	GBK-041449	KENYA
281	IT327003	GBK-041499	TURKEY
282	IT331859	KSL 170122	KOREA

Table S2. Distribution of agronomic trait and fatty acid content data from 282 sesame accessions.

**Agronomic traits**	**Class interval**	**Class**	**Frequency**
Days to flowering (DTF)	10	41-51	98
		51-61	88
		61-71	58
		71-81	31
		81-91	4

		91-101	1
		101-111	0
		111-121	2
Number of capsules per plant (NCP)	109	92-201	14
		201-310	58
		310-419	83
		419-528	67
		528-637	33
		637-746	14
		746-855	7
		855-964	5
Height of the first capsule-bearing node (HFC)	23	17-40	43
		40-63	79
		63-86	46
		86-109	41
		109-132	42
		132-155	22
		155-178	6
		178-201	3
Capsule zone length (CZL)	17	17-34	4
		34-51	26
		51-68	47
		68-85	42
		85-102	51
		102-119	60
		119-136	43
		136-153	9
Days to maturity (DTM)	9	85-94	16
		94-103	14
		103-112	46
		112-121	81
		121-130	88

		130-139	24
		139-148	10
		148-157	2
Capsule length (CL)	0.25	2.00-2.25	3
		2.25-2.50	22
		2.50-2.75	66
		2.75-3.00	110
		3.00-3.25	59
		3.25-3.50	18
		3.50-3.75	2
		3.75-4.00	2
Capsule width (CW)	0.11	0.50-0.61	2
		0.61-0.72	80
		0.72-0.83	113
		0.83-0.94	54
		0.94-1.05	29
		1.05-1.16	1
		1.16-1.27	1
		1.27-1.38	2
1,000-seed weight (1,000-SW)	0.4	1.00-1.40	1
		1.40-1.80	2
		1.80-2.20	5
		2.20-2.60	23
		2.60-3.00	58
		3.00-3.40	115
		3.40-3.80	71
		3.80-4.20	7
Location of branching (LB)	-	Lower	168
		Middle	109
		Upper	3
Number of capsules per axil (NCPA)	-	Single capsule	228
		Three capsules	54
Number of locules per capsule (NLPC)	-	Four locules	276

		Eight locules	1
		Mixture 8 and 6	1
		Mixture 4 and 6	2
		Mixture 4 and 8	2
Seed coat color (ScC)	-	White	133
		Light brown	51
		Brown	64
		Black	23
		Olive	11
Capsule hairiness (CH)	-	Glabrous	112
		Weak	1
		Medium	154
		Strong	15
Oil-related traits	Class interval	Class	Frequency
Total oil content (TOC)	3.5	34.00-37.50	2
		37.50-41.00	3
		41.00-44.50	9
		44.50-48.00	58
		48.00-51.50	95
		51.50-55.00	99
		55.00-58.50	16
		58.50-62.00	0
Myristic acid (C14:0)	0.01	0.00-0.01	28
		0.01-0.02	0
		0.02-0.03	222
		0.03-0.04	32
		0.04-0.05	0
		0.05-0.06	0
		0.06-0.07	0
		0.07-0.08	0
Palmitic acid (C16:0)	0.4	7.4-7.8	5
		7.8-8.2	27
		8.2-8.6	46

		8.6-9.0	77
		9.0-9.4	86
		9.4-9.8	36
		9.8-10.2	5
		10.2-10.6	0
Palmitoleic acid (C16:1)	0.01	0.08-0.09	1
		0.09-0.10	25
		0.10-0.11	52
		0.11-0.12	73
		0.12-0.13	78
		0.13-0.14	34
		0.14-0.15	16
		0.15-0.16	3
Stearic acid (C18:0)	0.25	3.70-3.95	6
		3.95-4.20	31
		4.20-4.45	87
		4.45-4.70	79
		4.70-4.95	48
		4.95-5.20	19
		5.20-5.45	10
		5.45-5.70	2
Oleic acid (C18:1)	2.5	29.6-32.1	26
		32.1-34.6	42
		34.6-37.1	81
		37.1-39.6	68
		39.6-42.1	27
		42.1-44.6	32
		44.6-47.1	4
		47.1-49.6	2
Linoleic acid (C18:2)	2.5	36.7-39.2	2
		39.2-41.7	15
		41.7-44.2	21
		44.2-46.7	55

		46.7-49.2	73
		49.2-51.7	71
		51.7-54.2	32
		54.2-56.7	13
Oleic acid/linoleic acid ratio (O/L ratio)	0.1	0.52-0.62	34
		0.62-0.72	66
		0.72-0.82	82
		0.82-0.92	56
		0.92-1.02	21
		1.02-1.12	19
		1.12-1.22	3
		1.22-1.32	1
Linolenic acid (C18:3)	0.1	0.20-0.30	83
		0.30-0.40	192
		0.40-0.50	5
		0.50-0.60	1
		0.60-0.70	0
		0.70-0.80	0
		0.80-0.90	0
		0.90-1.00	1
Arachidic acid (C20:0)	0.05	0.40-0.45	1
		0.45-0.50	63
		0.50-0.55	171
		0.55-0.60	40
		0.60-0.65	6
		0.65-0.70	0
		0.70-0.75	1
		0.75-0.80	0
Behenic acid (C22:0)	0.02	0.08-0.10	1
		0.10-0.12	32
		0.12-0.14	160
		0.14-0.16	69
		0.16-0.18	15

		0.18-0.20	4
		0.20-0.22	1
		0.22-0.24	0
Lignoceric acid (C24:0)	0.02	0.04-0.06	5
		0.06-0.08	205
		0.08-0.10	65
		0.10-0.12	6
		0.12-0.14	1
		0.14-0.16	0
		0.16-0.18	0
		0.18-0.20	0

Table S3. Eigenvalues and proportion of seven principal components to 20 traits of 282 sesame accessions.

Principal component	Eigenvalue	Proportion of variance (%)	Cumulative proportion (%)
1	2.46	30.30	30.30
2	1.70	14.47	44.78
3	1.50	11.25	56.03
4	1.06	5.58	61.61
5	1.01	5.09	66.70
6	0.96	4.63	71.33
7	0.95	4.49	75.82

Table S4. Correlation between the O/L ratio and agronomic traits in 23 screened sesame accessions.

Accession	Correlation between traits[z] Positive	Negative
IT029100	C18:0, C18:1, CW	C18:2, C16:0, C16:1, C22:0, DTM, HFC
IT029416	C18:0, C18:1, CW	C18:2, DTM
IT103957	C18:0, C18:1	C18:2, C14:0, C16:0, C16:1, C22:0, NCP, DTM, 1,000-SW
IT167133	C18:1, CL, CZL	C18:2, C18:3, C20:0, C22:0, C24:0, DTM, HFC
IT169293	C18:0, C18:1	C18:2, DTM, C16:0, C16:1, 1,000-SW, NCP
IT207353	C18:0, C18:1, C20:0	C18:2, DTM, C16:0, C16:1, NCP
IT209668	C18:0, C18:1,	C18:2, HFC
IT218015	C18:0, C18:1, C18:3, C20:0, CW	C18:2, C16:1
IT265176	C18:1, C14:0, CW	C18:2, C16:0, DTM
IT271254	C18:0, C18:1, CL	C18:2, DTM, C16:0, HFC
K276845	C18:0, C18:1, C20:0, C24:0, NCP	C18:2, DTM, 1,000-SW
IT310135	C18:0, C18:1, C20:0, C24:0, NCP	C18:2, DTM, 1,000-SW
IT318642	C18:0, C18:1, C20:0, C22:0, C24:0	C18:2, C16:1, DTM, NCP, TOC
IT318651	C18:0, C18:1, C20:0, C22:0	C18:2, DTM, NCP, 1,000-SW
IT331859	C18:0, C18:1, C24:0	C18:2, DTM, 1,000-SW
IT184346	C18:1, CZL	C18:0, C18:2, C16:0, C20:0, C22:0, HFC, DTF, DTM, NCP
IT194357	C18:1, CZL, CL	C18:0, C18:2, C20:0, C22:0, C24:0, DTF, DTM, HFC
IT201446	C18:1, 1,000-SW, TOC	C18:0,C18:2, C20:0, C22:0, C24:0, DTF, DTM, HFC
IT201448	C18:1, TOC, 1,000-SW	C18:2, C20:0, C22:0, C24:0, DTF, DTM, HFC,

IT201449	C18:1, C16:1, CL, 1,000-SW, TOC	C18:2, C18:3, C20:0, DTF, DTM, HFC, CW
IT201452	C18:1, C16:1, CL, 1,000-SW, TOC, NCP	C18:0, C18:2, C18:3, C20:0, C22:0, C24:0, DTF, DTM, HFC
K276873	C18:0, C18:1, C20:0	C16:0, C18:2, C22:0,
K276874	C18:0, C18:1, C20:0, TOC	C18:2, CL

[z]C16:0 : palmitic acid, C16:1 : palmitoleic acid, C18:0 : stearic acid, C18:1 : oleic acid, C18:2 : linoleic acid, C20:0 : arachidic acid, C22:0 : behenic acid, C24:0 : lignoceric acid, CW : capsule width, CL : capsule length, NCP : number of capsules per plant, CZL : capsule zone length, 1,000-SW : 1,000-seed weight, TOC : total oil content, DTF : days to flowering, DTM : days to maturity, HFC : height of the first capsule-bearing node.

Table S5. Analysis of Variance (ANOVA) of the O/L ratio across *FAD2* haplotypes in sesame accessions.

**Source**	**DOF**[z]	**SS**[y]	**MS**[x]	**F value**	***p***
Haplotype	2	1.2811	0.6405	153	7.55E-13
Residuals	20	0.0837	0.0042		
Haplotype	O/L ratio	SD[w]	Max[v]	Min[u]	Significance
Hap1	1.0755	0.0754	1.3199	1.0058	a
Hap2	0.5834	0.0266	0.6034	0.5325	b
Hap3	0.5700	0.0246	0.5784	0.5527	b

[z]DOF: degree of freedom; [y]SS: sum of square; [x]MS: mean sum of square; [w]SD: standard deviation; [v]Max: Maximum of O/L ratio value, [u]Min: Minimum of O/L ratio value. The same letter in significance column are not significantly different at 5% level by Duncan Multiple Range Test (DMRT).
